# Long-Term Oncologic Outcomes of Laparoscopic versus Open Surgery for Middle and Lower Rectal Cancer

**DOI:** 10.1371/journal.pone.0135884

**Published:** 2015-09-03

**Authors:** Shaotang Li, Feizhao Jiang, Jingfu Tu, Xiaofeng Zheng

**Affiliations:** Department of General Surgery, the First Affiliated Hospital of Wenzhou Medical University, Wenzhou, Zhejian, People’s Republic of China; The University of Hong Kong, CHINA

## Abstract

**Background:**

Laparoscopic surgery for middle and lower rectal cancer remain controversial because anatomical and complex surgical procedures specifically influence oncologic outcomes. This study analyzes the long-term outcomes of laparoscopic versus open surgery for middle and lower rectal cancer.

**Methods:**

Patients (laparoscopic: n = 129, open: n = 152) who underwent curative resection for middle and lower rectal cancer from 2003 to 2008 participated in the study. The same surgical team performed all operations. The mean follow up time of all patients was 74.3 months.

**Results:**

No statistical difference in local recurrence rate (7.8% vs. 7.2%; log-rank = 0.024; *P* = 0.876) and distant recurrence rate (20.9% vs.16.4%; log-rank = 0.699; *P* = 0.403) between laparoscopic and open groups were observed within 5 years. The 5-year overall survival rates of the laparoscopic and open groups were 72.9% and 75.7%, respectively; no significant statistical difference was observed between them (log-rank = 0.163; *P* = 0.686). The 5-year survival rates between groups were not different between stages: Stage I (92.6% vs. 86.7%; log-rank = 0.533; *P* = 0.465); stage II (75.8% vs. 80.5%; log-rank = 0.212; *P* = 0.645); and Stage III (63.8% vs. 69.1%, log-rank = 0272;P = 0.602). However, significant statistical difference amongst different stages were observed (log-rank = 1.802; *P* = 0.003).

**Conclusion:**

Laparoscopic and open surgery for middle and lower rectal cancer offer equivalent long-term oncologic outcomes. Laparoscopic surgery is feasible in these patients.

## Introduction

Recent published reports of randomized trials that Laparoscopic resection (LR) for colorectal cancer is a feasible and safe technology [1–6]. Some randomized trials have demonstrated that laparoscopic surgery for colorectal cancer provides equivalent oncologic outcomes and better short-term outcomes when compared to open surgery[7–11]. LR for middle and lower rectal cancer surgery, however, remains controversial mainly because of the steep learning curve, technical challenges (e.g., difficulties in pelvic exposure and sphincter preservation, and preservation of the autonomic nerves during performing total mesorectal excision (TME), [12–18]). There also exists a lack of long-term data from large-scale studies that may be used to evaluate the procedure [19, 20]. Researchers today are particularly eager to determine whether the laparoscopic technique can truly achieve adequate tumor clearance and has oncologic outcomes similar to those of open resection (OR). The aim of the present comparative, prospective study is to assess the long-term oncologic outcomes of laparoscopic versus open surgery for middle and lower rectal cancer. The study is a unicentric comparative series that includes more than 100 curative middle and lower rectal laparoscopic resections and more than 10 years’ worth of results. The largest for any published study on laparoscopic versus open surgery that specifically addresses middle and lower rectal cancer.

## Materials and Methods

### Patients

This research had been approved by Institutional Review Board (IRB) of the First Affiliated Hospital of Wenzhou Medical University. Patients with middle and lower rectal cancer (the distance of tumor from anal verge is within 10 cm) undergoing curative resection were invited to participate in the study before their treatment from January 2003 to December 2008. Laparoscopic and open surgery procedures were considered at the same standard-of-care for middle and lower rectal cancer at our institution.Patients chose inclusion to the laparoscopic or open groups based on the current stage of their disease and after understanding the risks and benefits inherent in laparoscopic and open resections by themselves without any pressure from the surgeon. All patients provided written informed consent. Patients, who underwent emergent surgery, palliative resection or bypass, or transanal resection, or intersphincteric resection, were excluded from the study. Patients with evidence of synchronous metastatic disease were also excluded.

### Preoperative Examination

All patients underwent physical examination, total colonoscopy plus biopsy, rigid sigmoidoscopy, anorectal ultrasonography, thoracic and abdominal computed tomography (CT), and pelvic magnetic resonance imaging (MRI). Patients without a complete colonoscopy were administered a barium enema. Regular preoperative blood tests, including a complete blood count, a blood chemistry test, and a serum carcinoembryonic antigen (CEA) determination, were performed on all patients.

### Neoadjuvant Chemoradiotherapy

The basic indications for neoadjuvant chemoradiotherapy included full-thickness rectal cancers (T3 or T4) by MRI or anorectal ultrasonography and/or node-positive disease, lack of evidence of distant metastases, lack of prior radiation therapy to the pelvis, and patient age ≤75 years. Neoadjuvant treatment with chemotherapy and radiation therapy was as follows: 50 Gy in 5 weeks with concomitant 5-fluorouracile-based schedule throughout the study. The operation was carried out 4–6 weeks after the end of the neoadjuvant treatment.

### Surgical Technique

All operations were performed by the same surgical team, including X.Z, F. J, and J. T, all of whom have had experience in open TME and advanced laparoscopic colorectal surgery. All patients had bowel preparations, including a fluid diet and administration of a polyethylene glycol electrolyte solution, one day before the operation unless there were contraindications against bowel preparation. Intravenous antibiotic prophylaxis was given on induction of anesthesia for the operation.

All patients underwent TME with preservation of the pelvic nerves. An abdominoperineal resection (APR) was performed when the tumor infiltrated the anal canal or when it was impossible to obtain a distal margin of more than 1 cm. For anterior resection (AR), stapled end-to-end colorectal or hand-sewn coloanal anastomoses were constructed. Patients undergoing open surgery were placed in the Lloyd-Davis position, and the abdominal and pelvic cavity was accessed via a midline laparotomy extending from above the umbilicus to the pubis. Laparoscopic surgery procedure was similar to that of professor Chi[21]. Patients who underwent protective colostomy were mainly those who underwent neoadjuvant treatment and/or with anastomotic stoma distance of within 3 cm from the anal verge in AR. Other patients underwent colorectal decompression via a 1.5cm diameter anal drainage tube in AR for 5-7 days. Conversion to open surgery was decided upon inability to complete the laparoscopic resection.

### Postoperative Care

A standardized postoperative management protocol was established. The nasogastric tube was removed at or before 24 hours. The urinary catheter was left in place until the day after bladder function recovery, except in cases of known or suspected prostatic disease. Oral feeding was started as soon as the return of intestinal function was confirmed. Patients were discharged after all drains had been removed; discharged patients were with afebrile and able to tolerate oral intake. After laparoscopy and open surgery, patients instage II/III received postoperative adjuvant chemotherapy with 5-fluorouracil, leucovorin and oxaliplatin (FOLFOX) for 6 months.

### Follow Up

All the patients were followed up prospectively with history and clinical examinations, a serum CEA assay were performed every 3 or 6 months for 2 years, and then every 6 months for a total of 5 years. Chest, abdominal and pelvic CT scans and proctoscopy for patient status AR were performed every 6 months for 2 years, annual follow-ups followed thereafter. A colonoscopy was performed annually for up to 5 years. If recurrence was suspected at any time after the operation, a CT was performed. Data were collected prospectively from the time of diagnosis using a custom-written computerized database. The last follow up was in December 2013.

### Measured Outcomes and Definitions

Blood loss was evaluated intraoperatively using accepted techniques, including weighing of gauzes at the end of the operation. The pathologic specimen was evaluated by experienced pathologists through a standardized method. The histologic grade, presence of lymph node metastasis, and lymphovascular or neural invasion were evaluated. A complete TME means that the mesorectum was intact with only minor irregularities in its smooth mesorectal surface, no defect was deeper than 5 mm, and no coning toward the distal margin of the specimen was observed. A smooth circumferential resection margin was obtained at slicing. R0 was defined as a complete tumor resection with all margins histologically negative. R1 was defined as an incomplete tumor resection with microscopic surgical resection margin involvement (margins grossly uninvolved). An anastomotic leakage was defined as clinical staple line leaks, infectious collections in the pelvis (with or without a proven staple line leak), or anenterocutaneous or vaginal fistula. Morbidities were defined as complications that required additional treatment or prolonged hospital stay. Operative mortality was defined as death within 30 days after operation. Survival time was calculated from the date of surgery.

### Statistical Analysis

Data were analyzed using the intention-to-treat (ITT) principle. Conversion patients remained in the laparoscopic group. Normal distribution data were described by mean ± standard deviation ($\bar{x}$ ±s) and analyzed by t-tests. Non-normal distribution data were described by the median and range and analyzed through the Mann–Whitney U test. Numeration data were analyzed by either the chi-square test or Fisher exact test where appropriate. Data normality was analyzed using the Kolmogorov-Smirnov test. The cumulative risk of the 5-year recurrence (local recurrence and distant recurrence) and the 5-year overall survival were calculated using the Kaplan–Meier method and compared by log-rank test. All statistical analyses were performed using SPSS 17.0 (Chicago, IL). A *P* value of 0.05 was determined to be significant.

## Results and Discussion

**Fig 1:**
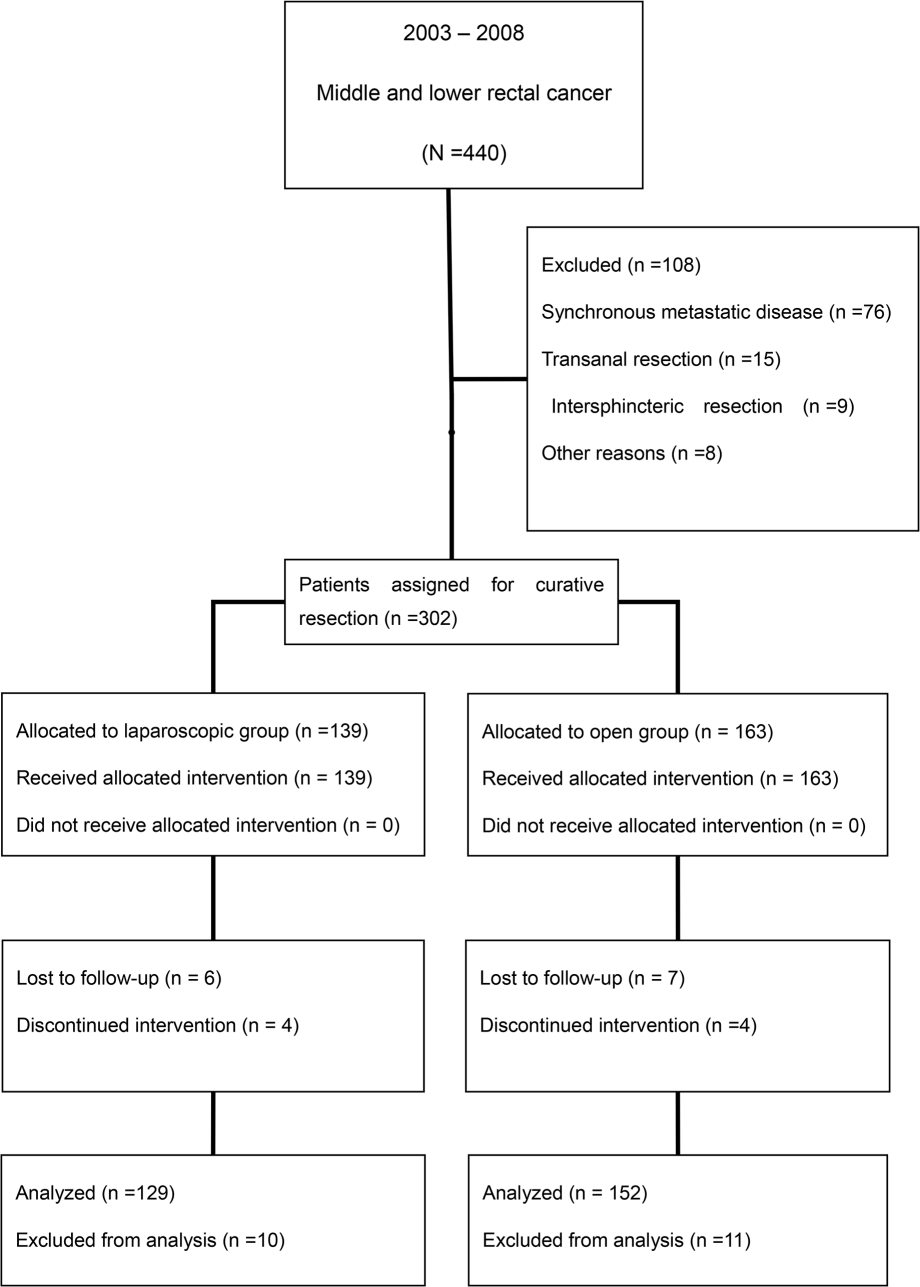
Patients’ selective procedure.

Four hundred and forty patients enrolled in the present study; 281patients were included in the analysis, 129 patients underwent LR, and 152 patients underwent OR (Fig 1). A comparison of patient details is shown in Table 1. No significant differences were noted between laparoscopic and open patients (*P* > 0.05). For all patients, the mean follow up was 74.3 months, ranging from 1–131 months.

**Table 1:** Patient details.

	Laparoscopic	Open	
	(n = 129)	(n = 152)	*P* value
Age (years)	56.2 ± 13.5	57.6 ± 14.0	0.402
Sex			0.442
Male	78 (55.9%)	85 (60.5%)	
Female	51 (44.1%)	67 (39.5%)	
BMI (kg/m^2^)	22.2 ± 2.3	22.1 ± 2.3	0.486
ASA			0.236
1	77 (59.7%)	76 (50.0%)	
2	42 (32.6%)	64 (42.1%)	
3	10 (7.8%)	12 (7.9%)	
Location of tumor			0.436
Middle rectum (5.1–10.0 cm)	97 (75.2%)	108 (71.1%)	
Lower rectum (0–5.0 cm)	32 (24.8%)	44 (28.9%)	
T category			0.728
T1	16 (12.4%)	10 (6.6%)	
T2	20 (15.5%)	25 (16.4%)	
T3	21 (16.3%)	27(17.9%)	
T4	72 (55.8%)	90 (59.2%)	
N category			0.310
N0	60 (46.5%)	71 (46.7%)	
N1	41 (31.8%)	38 (25.0%)	
N2	28 (21.7%)	43 (28.3%)	
pTNM stage			0.799
I	28 (21.7%)	29 (19.1%)	
II	32 (24.8%)	42(27.6%)	
III	69 (53.5%)	81 (53.3%)	
Tumor differentiation ^a^			0.488
Well	45 (34.9%)	42 (27.6%)	
Moderate	64(49.6%)	78 (51.3%)	
Poor	19 (14.7%)	30 (19.7%)	
Undifferentiated	1 (0.8%)	2 (1.3%)	
Neoadjuvant chemoradiotherapy	48 (36.4%)	55(36.2%)	0.965
Postoperative chemotherapy	75 (58.1%)	84 (55.3%)	0.628
Follow-up (months)	74.8± 31.2	73.9± 31.7	0.815

^a^ Fisher exact test. ASA, American Society of Anesthesiologists Class; pTNM, tumor, node and metastasis; BMI, body mass index

### Operative and Postoperative Outcomes

Eight patients were converted to open procedures, five because of severe adhesion, two because of failure to control massive bleeding and one because of difficulties in pelvic exposure, corresponding to a conversion rate of 6.2%. Operative and postoperative outcomes are summarized in Table 2. No differences (*P* > 0.05) were detected in surgical procedures (AR or APR), operative time, protective colostomy, lymph nodes harvested, distal resection margins, resection margin involvement (R0 or R1), and complete TME. The operative blood loss in the laparoscopic group was significantly less than in the open group (*P* = 0.000). Postoperative morbidities were 27 (20.9%) for the laparoscopic group and 42 (27.6%)for the open group, it was no differences (*P* > 0.05). Incidence of anastomotic leakage, which was the most common postoperative morbidity, was 7.1% vs. 8.9%. In the laparoscopic group, one patient died of heart failure 3 days post-operation and one patient died of abdominal infection 28 days post-operation. In the open group, one patient died of multiple organ failure (MOF) 16 days post-operation, one patient died of abdominal infection 24 days post-operation. There was significant statistical difference in the length of stay between laparoscopic and open groups (*P* = 0.000).

**Table 2:** Operative and postoperative outcomes.

	Laparoscopic	Open	
	(n = 129)	(n = 152)	*P* value
Surgical procedure			0.128
Anterior resection	113 (80.9%)	123 (78.6%)	
Abdominoperineal resection	16(19.1%)	29 (12.4%)	
Protective colostomy	28(21.7%)	33 (21.7%)	0.999
Operative time (min)	177.9± 50.5	170.7 ± 62.3	0.289
Blood loss (ml)	50.0(0–1000)	150.0(0–1200)	0.000
Lymph nodes harvested	20.2 ± 7.1	20.2 ± 8.3	0.994
Distal resection margin (cm)	3.3 ± 1.3	3.3 ± 1.3	0.888
Resection margin involvement			0.558
R0	123 (95.3%)	147 (96.7%)	
R1	6 (4.7%)	5 (3.3%)	
Complete TME	118 (91.5%)	141 (93.4%)	0.536
Reoperations	10(7.8%)	12(7.9%)	0.965
Morbidity	27(20.9%)	42 (27.6%)	0.193
Anastomotic bleeding	4 *(3*.*1%)*	6 (3.9%)	
Anastomotic leakage ^a^	*8 (7*.*1%)*	11 (8.9%)	
Wound bleeding	1 *(0*.*8%)*	2*(1*.*3%)*	
Wound infection	3 (2.3%)	4 (2.6%)	
Perineal infection	0(0.0%)	1 (0.7%)	
Abdominal infection	*(1*.*6%)*	3 (2.0%)	
Urinary retention	3 (2.3%)	5 (3.3%)	
Urinary infection	1 *(0*.*8%)*	2*(1*.*3%)*	
Bowel obstruction	2 *(1*.*6%)*	4 (2.6%)	
Respiratory infections1	*(0*.*8%)*	1 (0.7%)	
Others	2 *(1*.*6%)*	3 (2.0%)	
Perioperative mortality ^b^	2 *(1*.*6%)*	2 (1.3%)	0.625
length of stay (days)	9.0 (6–48)	10.0 (7–51)	0.000

^a^ Patients undergoing abdominoperineal resection were excluded,

^b^Fisher exact test.

### Long–Term Outcomes

**Fig 2:**
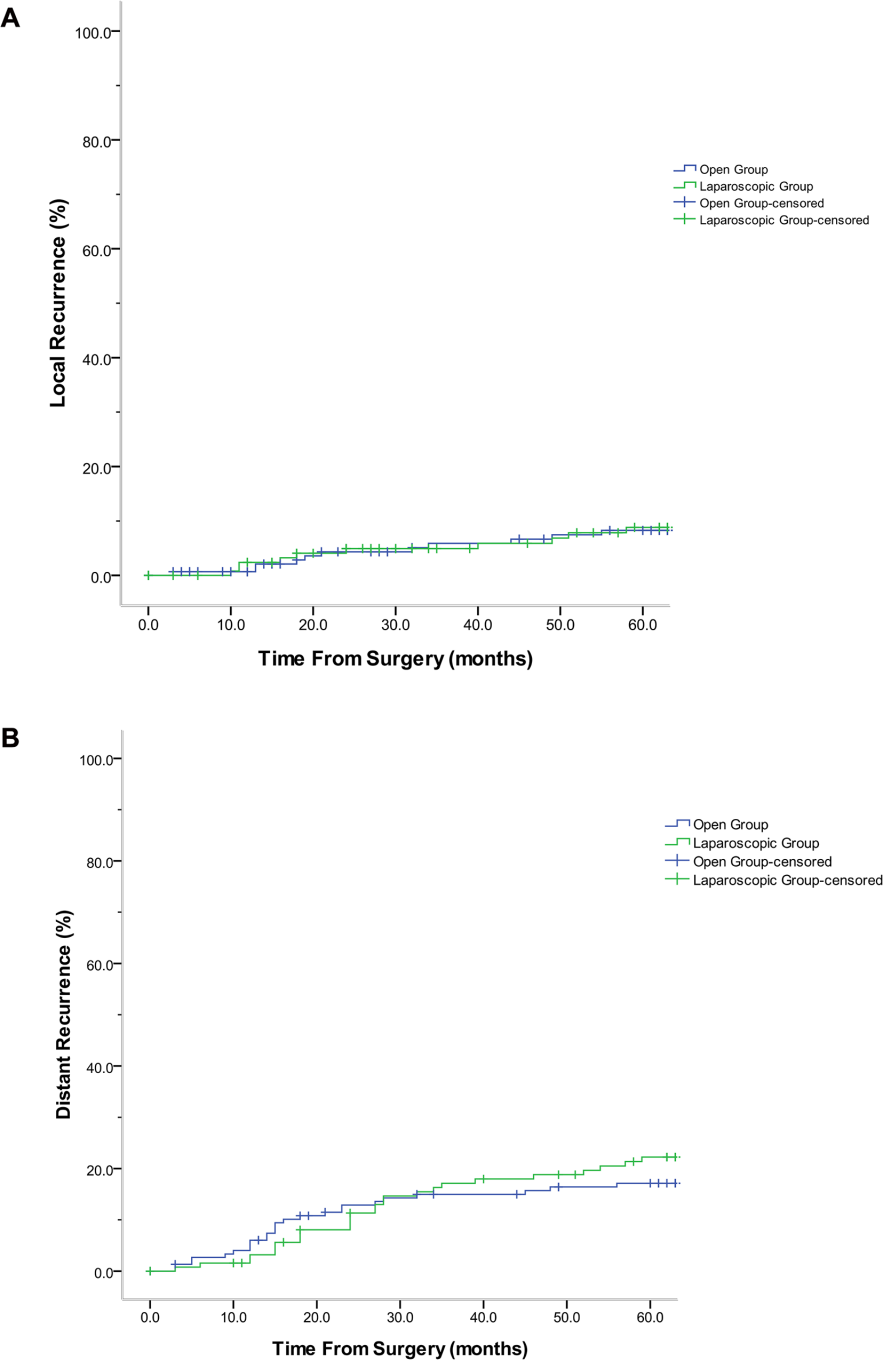
A. Comparison of local recurrence of patients between laparoscopic and open groups (log-rank = 0.432; *P* = 0.511). No. at risk: Laparoscopic group: 111.5\104.5\95.0\89.0\85.5\41.5; Open group: 120.5\111.5\100.5\97.5\96.0\47.0. B. Comparison of distant recurrence of patients between laparoscopic and open groups (log-rank = 0.505; *P* = 0.477). No. at risk: Laparoscopic group: 111.5\107.0\98.0\89.5\85.5\41.5; Open group: 121.5\114.0\101.5\98.0\96.5\47

**Fig 3:**
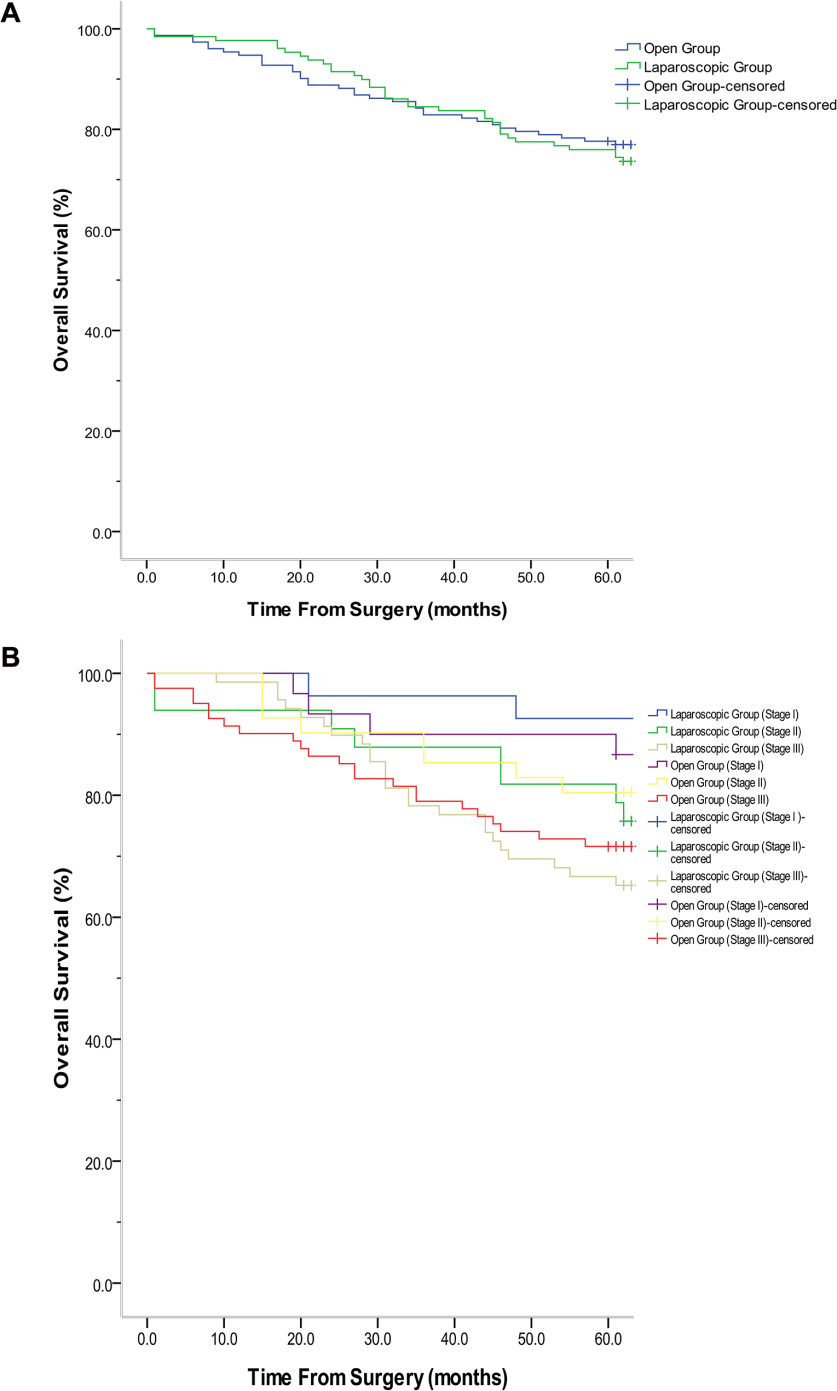
A. Comparison of the 5-year overall recurrence of patients between laparoscopic and open groups (log-rank = 0.012; *P* = 0.913,). No. at risk: Laparoscopic group: 113.0\111.0\106.0\97.0\91.0\45.0;Open group: 123.0\119.0\110.0\104.0\100.0\49.5. B. Comparison of the 5-year overall survival in patients of different stages between laparoscopic and open group (P = 0.004).

Fifty-seven cases had cancer recurrence within 5 years, with the liver being the most common site of recurrence. Of the 73, 25(8.9%) cases were detected in the liver as the first site of recurrence. No wound or port-site recurrence was detected in either group. Local recurrence rate (7.8% vs. 7.2%; log-rank = 0.024; *P* = 0.876) and distant recurrence rate (20.9% vs.16.4%; log-rank = 0.699; *P* = 0.403) between laparoscopic and open groups were observed within 5 years, no significant statistical difference was observed between them(Fig 2). The 5-year overall survival rates of the laparoscopic and open groups were 72.9% and 75.7%, respectively; no significant statistical difference was observed between them (log-rank = 0.163; *P* = 0.686; Fig 3A).The 5-year overall survival rates of the all patents were 74.4%. The 5-year overall survival rate among patients of different stages in the laparoscopic and open groups was studied. Overall survival curve were shown in Fig 3B. The 5-year survival rates between groups were not different between stages: Stage I (92.6% vs. 86.7%; log-rank = 0.533; *P* = 0.465); Stage II (75.8% vs. 80.5%; log-rank = 0.212; *P* = 0.645); and Stage III (63.8% vs. 69.1%, log-rank = 0272; P = 0.602). However, significant statistical difference amongst different stages were observed (log-rank = 1.802; *P* = 0.003).

Laparoscopic surgery yields more cosmetically appealing incisions, less analgesic requirements, and earlier return of patients’ functionalities. Although several randomized trials [3,7, 9, 11, 18–20,22–23] that compare laparoscopic and open colectomy for colon cancer in terms of equivalent recurrence and survival rate are well underway, the number of prospective randomized trials that aim to evaluate laparoscopic resection for rectal cancer is limited [4, 10, 24–30]. Of those available, only four specifically address middle and lower rectal cancer [10, 26–28], and only one reported its long-term (5 years) outcomes in a small sample trial [27]. There are different biological behaviors and clinical outcomes of cancers located between the upper rectum and the middle or lower rectum. Analysis of long-term survival is essential to evaluate the oncologic efficacy of any cancer treatment. Because of the lack of long-term (5 years) data on survival and recurrence, the role of laparoscopy in middle and lower rectal cancer resection is heatedly debated.

This study compares the long-term outcomes after laparoscopy with open surgery for middle and lower rectal cancer. The largest for any published study on laparoscopic versus open surgery that specifically addresses middle and lower rectal cancer.

In laparoscopic surgery, the major challenge that quickly became apparent in the randomized trials of the 1990s was the high rate of conversion, ranging from 11% in the Barcelona trial to 29% in the UK MRC CLASICC trial [7, 8]. In the rectal cancer cohort of the CLASICC trial, the rate of conversion was even higher at 34%. Conversion rates for the laparoscopic resection of rectal cancer have been recently reported to range between 2.8% and 9.8% [14, 28, 30, 31]. Our conversion rate of 6.2% is comparable to the conversion rate of 5.4% in Hong Kong trials. The low conversion rate reflects the importance of an accumulation of experience and a specialized team. The present study shows that there are similar qualities of surgery, e.g., complete TME, RO resection, lymph nodes harvesting, and distal resection margin, which indicate that LR could satisfy radical resection [32–36]. Many studies, including the present one, have found laparoscopic surgical resection to be associated with significantly longer operating time compared to the open equivalent [4, 8, 18], but, our study shows that there is similar operating time.

Laparoscopic surgical resection for rectal cancer also offered wider and clearer vision into the narrow pelvic cavity, which is advantageous for the preservation of the autonomic nerves and TME. Blood loss and blood product requirements in laparoscopic compared to open surgery are unclear. Some studies estimated reduced blood loss [8, 37]. Whereas others suggested that the blood loss was comparable [23]. Patients who underwent LR in the present study had less blood loss than those who underwent OR. Decreasing costs of stored blood, together with the laboratory costs associated with cross-matching, have obvious financial implications in favor of laparoscopic surgery. Functionalities among patients in the laparoscopic group returned earlier; their mean length of stay in the hospital was significantly shorter than that of the open group [38,39], it similar to present study.

There were no differences in the 5-year recurrence rate (local recurrence and distant recurrence) between laparoscopic and open groups, and no wound and port-site recurrence was detected, similar to other reports [9, 30], because the laparoscopic and open surgical techniques strictly followed the oncologic principles of tumor resection.

Although two series reported a higher survival rate because of the laparoscopic approach after colorectal surgery [40,41], there was no difference in the 5-year overall survival rate between laparoscopic and open surgery in the present study. The 5-year overall survival rate (72.9%) in the laparoscopic group was comparable to similar reports that estimated them to be at 75.2% [30] and 73.7% [31]and 77.9%[21]. This indicates that LR was not inferior to OR in terms of qualities of surgery. Moreover, the present study shows that there was a similarity in the 5-year overall survival rate in patients from different stages between laparoscopic and open groups. In the laparoscopic group, the survival rate was 92.6% in Stage I, 75.8% in Stage II, and 63.8% in Stage III. These rates are comparable to similar reports of 91%, 82%, and 56% for rectal cancer [30], respectively. Laparoscopic surgery is similar to open surgery for patients from any stage of rectal cancer.

The choice of operation method is not a randomized process in the present study. As a result, there may be selection bias. However, the results are consistent because the characteristics of the patients in the two surgery groups were not significantly different. The present study confirms the feasibility of laparoscopic surgery for middle and lower rectal cancer. Although these results were obtained from a team that specialized in both laparoscopic and open surgery approaches and operated on a high volume of cases, laparoscopic surgery should become a standard in selected middle and lower rectal cancer cases in the future because of technological developments, specialization of surgeons, and the demonstrated advantages of the procedure.

## Conclusions

Laparoscopic and open surgery for middle and lower rectal cancer offer equivalent long-term oncologic outcomes. Laparoscopic surgery is feasible in these patients. Future randomized controlled trials are required to address long-term oncologic outcomes related to laparoscopic surgery for middle and lower rectal cancer.
